# Supplementary material for: Sonication-Assisted Method for Decellularization of Human Umbilical Artery for Small-Caliber Vascular Tissue Engineering
Source: Polymers (Basel). 2021 May 22;13(11):1699. doi: 10.3390/polym13111699 (PMC8196986; doi:10.3390/polym13111699)
Supplement: Supplementary file 1 [file polymers-13-01699-s001.zip › polymers-1203362-supplementary.pdf]

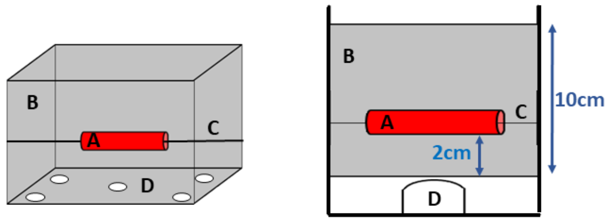

Figure S1. Schematic diagram of sonication-assisted decellularization. (**A**) Human umbilical artery; (**B**) Wash buffer; (**C**) Cotton thread; (**D**) Ultrasonic horn (Frequency:40 kHz, HF-peak out:407 Watt.).

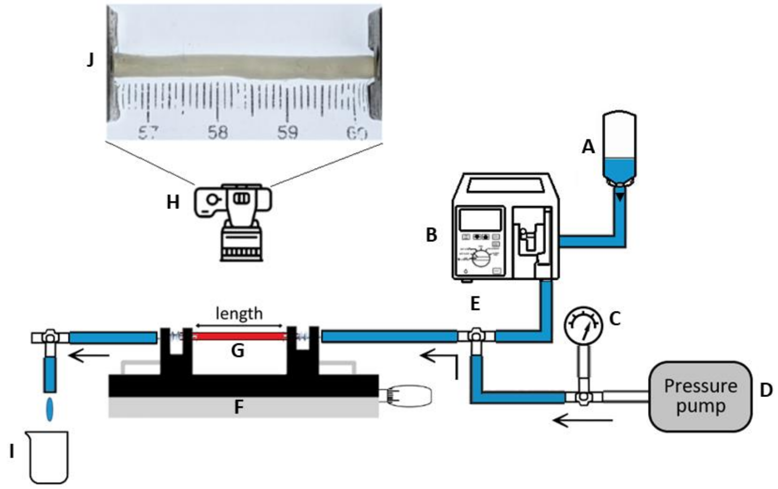

Figure S2. Mechanical property test device. (**A**) Lactated Ringer solution; (**B**) infusion pump; (**C**) Sphygmomanometer; (**D**) Pressure pump; (**E**) three-way valve; (**F**) customized mechanical device; (**G**) umbilical artery; (**H**) camera; (**I**) beaker; (**J**) Image of artery taken by camera (Arrow: air pressure flow, arrow head: fluid flow).

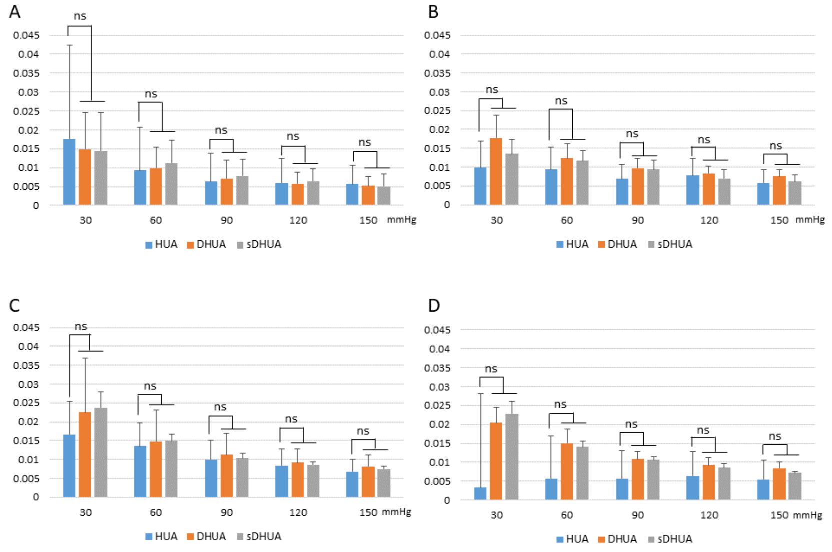

Figure S3. Mechanical property (compliance) between HUA, cDHUA, and sDHUA at different stretch ratio. λ = 1.0; (**B**) λ = 1.2; (**C**) λ = 1.3; (**D**) λ = 1.4; ns: no significance.

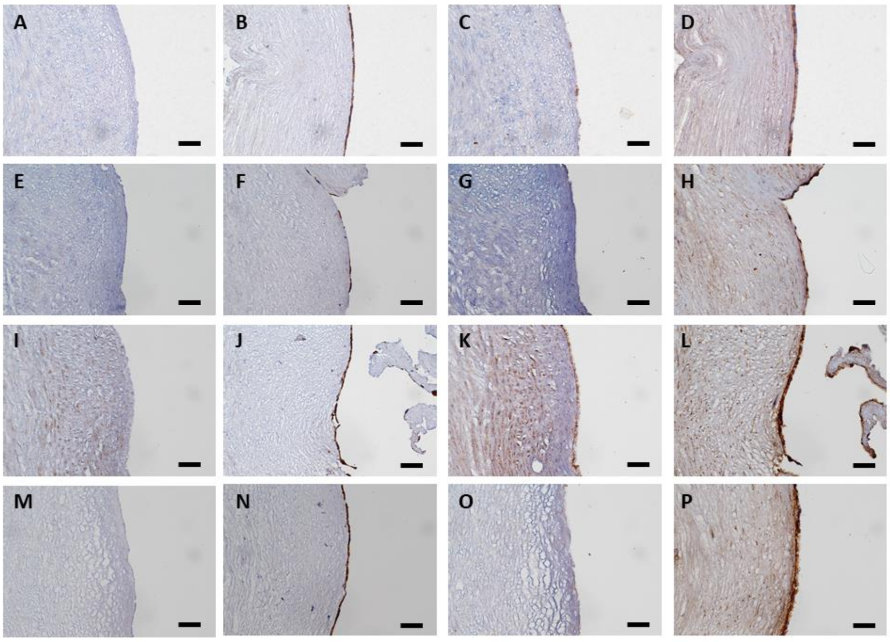

Figure S4. Histological stains of cell seeding on sDHUA. (**A)**,(**C**) sDHUA, 204 W for 4 h without seeding; (**B)**,(**D**) sDHUA, 204 W for 4 h with seeding; (**E)**,(**G**) sDHUA, 204 W for 8 h without seeding; (**F)**,(**H**) sDHUA, 204 W for 8 h with seeding; (**I)**,(**K**) sDHUA, 285 W for 4 h without seeding; (**J)**,(**L**) sDHUA, 285 W for 4 h with seeding; (**M)**,(**O**) sDHUA, 285 W for 8 h without seeding; (**N)**,(**P**) sDHUA, 285 W for 8 h with seeding; (**A)**,(**B)**,(**E)**,(**F)**,(**I)**,(**J)**,(**M)**,(**N**) CD31; (**C)**,(**D)**,(**G)**,(**H)**,(**K)**,(**L)**,(**O)**,(**P**) vWF; Magnification: 200 ×, Scale bar = 50 µm.
